# GPCR-Mediated Cell Intelligence: A Potential Mechanism for Survival and Long-Term Health

**DOI:** 10.3390/cimb48020127

**Published:** 2026-01-23

**Authors:** Carter J. Craig, Tabitha Boeringer, Mia Pardo, Ashley Del Pozo, Stuart Maudsley

**Affiliations:** Receptor Biology Laboratory, Department of Drug Discovery, Division of Basic Sciences, H. Lee Moffitt Cancer Center, Tampa, FL 33612, USA; carter.craig@moffitt.org (C.J.C.); tabitha.boeringer@moffitt.org (T.B.); adelpozo@usf.edu (A.D.P.)

**Keywords:** receptor, intelligence, cell, resilience, stress

## Abstract

The concept of individual cellular intelligence reframes cells as dynamic entities endowed with sensory, reactive, adaptive, and memory-like capabilities, enabling them to navigate lifelong metabolic and extrinsic stressors. A likely vital component of this intelligence system is stress-responsive G protein-coupled receptor (GPCR) networks, interconnected by common signaling adaptors. These stress-regulating networks orchestrate the detection, processing, and experience retention of environmental cues, events, and stressors. These networks, along with other sensory mechanisms such as receptor-mediated signaling and DNA damage detection, allow cells to acknowledge and interpret stressors such as oxidative stress or nutrient scarcity. Reactive responses, including autophagy and apoptosis, mitigate immediate damage, while adaptive strategies, such as metabolic rewiring, receptor expression alteration and epigenetic modifications, enhance long-term survival. Cellular experiences that are effectively translated into ‘*memories*’, both transient and heritable, likely rely on GPCR-induced epigenetic and mitochondrial adaptations, enabling anticipation of future insults. Dysregulation of these processes and networks can drive pathological states, shaping resilience or susceptibility to chronic diseases like cancer, neurodegeneration, and metabolic disorders. Employing molecular evidence, here, we underscore the presence of an effective cellular intelligence, supported by multi-level sensory GPCR networks. The quality of this intelligence acts as a critical determinant of somatic health and a promising frontier for therapeutic innovation. Future research leveraging single-cell omics and systems biology may unravel the molecular underpinnings of these capabilities, offering new strategies to prevent or reverse stress-induced pathologies.

## 1. Introduction

Cells, the fundamental units of life, can be viewed as mechanistic entities executing pre-programmed functions to ensure their ability to perform their tissue assigned and associated functions. Arguably one of the most fundamental functions of a cell—from any specific tissue—is just to survive and to continue to carry out their assigned functions during the organism’s lifespan. Cells possess an intrinsic drive to develop and proliferate at varying rates depending on the nature of the tissues, e.g., rapidly growing gut lining cells of slow growing pluripotent stem cells. Impeding this necessary cell growth and development is the ever-present but stochastic element of impinging damage and stress to the cell. Among the fundamental functions of cells across tissues is the survival and maintenance of assigned roles throughout an organism’s lifespan—often in the face of stochastic stressors whose timing and nature are unpredictable. While expected in principle, the nature, extent, and time of this stress are largely unknown to the cell. This ever-present facet of cell biology and disease etiology, i.e., stochastic damage, is a vital factor for the cell to mitigate and/or overcome, thus enabling long- or short-term growth, development and proliferation of the cell in order to age successfully. Emerging evidence is beginning to reveal the presence of a dynamic paradigm of cellular ‘intelligence,’ defined by cells’ ability to sense environmental stressors, execute reactive and predictive adaptive responses, and also to retain memories of past challenges. This cell-intrinsic intelligence enables survival amidst relentless metabolic and extrinsic insults—such as reactive oxygen species (ROS), DNA damage, or toxic compounds—through sophisticated molecular networks dedicated to preserving ongoing cellular fitness and health [1,2]. Stress-responsive G protein-coupled receptor (GPCR) networks, functionally integrated with organelles such as the nucleus, stress granules, and mitochondria, likely form a dynamic signaling network that, together, (i) detects stressors, (ii) coordinates responses, and (iii) encodes stress history [3,4,5,6,7]. Sensory mechanisms, including GPCR-mediated signaling, nuclear factor erythroid 2-related factor 2 (Nrf2) pathways, and DNA damage response (DDR) systems, prioritize threats with precision and moderated responses aimed to mitigate the extent and potential spread of molecular damage [8,9]. Reactive responses, such as autophagy and apoptosis, address immediate damage, while adaptive strategies, like metabolic rewiring and epigenetic reprogramming, enhance resilience to recurring and chronic stressors [10,11]. Cells may also exhibit short- and long-term memory-like functions, ranging from transient protein modifications to heritable epigenetic changes, enabling anticipation of future insults [12,13]. Dysregulation of these sensory and memory-forming processes can lock cells into pathological states, driving chronic diseases such as cancer, metabolic syndrome, and neurodegeneration [14]. Understanding the qualitative and quantitative nature of cellular intelligence requires the exploration of the evolutionary pressures that shaped its origins, its spatial localization within the cell, and its mechanistic components, including organelles, molecular functions, and dynamic feedback and assessment loops. By defining cells as intelligent agents, this perspective synthesizes the evolutionary, spatial, and mechanistic foundations of cellular intelligence, emphasizes the role of GPCR networks and molecular mechanisms, and proposes research directions to harness these insights for therapeutic innovation, illuminating how daily stressors translate into long-term health outcomes.

## 2. Defining Cell Intelligence (CI)

The concept of individual cellular ‘intelligence’ identifies cells as dynamic, autonomous entities capable of perceiving their environment, making reactive and adaptive decisions, and intentionally retaining memory of past stressors to enhance their adaptability to stochastic future stress events. We propose that a cell’s long-term survival, amidst relentless metabolic and extrinsic challenges such as oxidative stress, DNA damage, or environmental toxins, relies on its ability to sense threats, respond appropriately, and learn from a diverse range of both beneficial and deleterious experiences. Here, we synthesize molecular evidence supporting the role of GPCR networks in coordinating cellular stress detection, adaptive responses, and memory-like processes. These capabilities not only ensure immediate stress resilience but also shape long-term cellular fates, potentially mitigating the development of chronic diseases like cancer, neurodegeneration, or metabolic disorders. By exploring the interconnected molecular mechanisms and single-cell physiological systems underlying cellular intelligence, we can potentially better understand how daily stressors contribute to disease and identify novel therapeutic strategies to prevent the transition of cell health to cell disease.

## 3. The Origins of Cellular Intelligence (CI)

Cells possess intricate molecular systems—composed of synergistic enzymes, ion channels, organelles, and receptors—that function as sensory and perceptive mechanisms, enabling the detection and interpretation of environmental stressors. The vertical and horizontal integration of these systems results in the generation of pro-intelligent cellular signaling systems that approaches the level of a sentient individual entity. This autonomous cellular entity organizes this complex system through the generation of an intelligence system.

### 3.1. Evolution of CI

This emerging cellular intelligence likely resulted from evolutionary pressures that favored the survival of cells capable of sensing and adapting to dynamic challenges in their environment [15]. Primordial unicellular organisms faced intensely fluctuating conditions—nutrient scarcity, oxidative stress, or predation—driving the development of sensory and signaling systems [16]. With respect to the ability of a cell to sense its environment, it is arguable that one of the most important mediators of this fundamental facet of survival is GPCRs. For instance, Bacteriorhodopsin, a light-driven proton pump found in archaea such as *Halobacterium salinarum*, represents one of the earliest known seven-transmembrane repeat proteins, enabling energy production via proton gradients in harsh, high-salinity environments [17]. This function was crucial for cellular survival, acting as a primitive stress response mechanism by maintaining ion homeostasis and ATP synthesis under osmotic or oxidative stress, where traditional respiration might fail. Structurally, bacteriorhodopsin belongs to the microbial rhodopsin (MR) family, which shares a heptahelical transmembrane architecture with eukaryotic GPCRs. Evolutionary analyses suggest that bacteriorhodopsin-like MRs, particularly sodium-translocating variants (e.g., KR2 from *Krokinobacter eikastus*), served as progenitors to the first GPCR-like factors. These ancient (>2 billion years old) pumps likely transitioned from ion transport to sensory signaling through key adaptations: loss of the retinal chromophore (creating a ligand-binding pocket), stabilization by sodium ions, and helical rearrangements that enabled conformational changes for downstream signaling. This prokaryotic lineage gave rise to eukaryotic rhodopsins, such as visual rhodopsin (opsin) in animals, which reacquired retinal for light detection while retaining the 7-TM fold and G protein coupling.

### 3.2. CI Development in Diverse GPCRs

**Figure 1:**
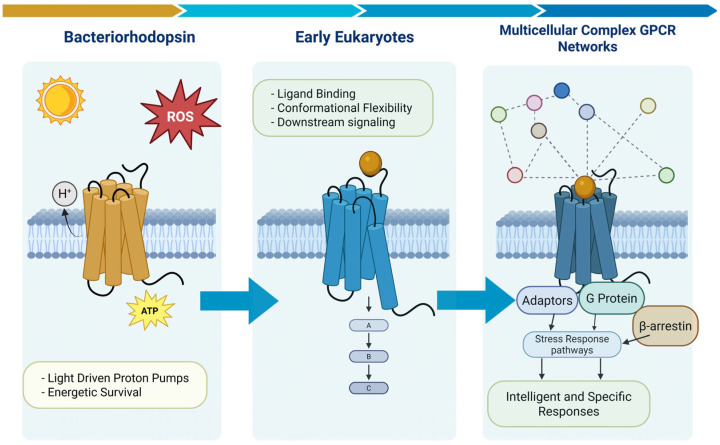
The evolution of GPCR signaling functionality. From the initial days of cellular life on earth, bacteriorhodopsin was able to protect early bacteria from stressful situations. This role of stress survival has continued to this day with the huge diversification of functional damage mitigation systems in today’s GPCR landscape.

Visual rhodopsin, a Class A (rhodopsin-like) GPCR, exemplifies this link, functioning in phototransduction for environmental sensing—a survival-critical adaptation for detecting light gradients or threats [18]. From there, diversification led to the broader rhodopsin-like GPCRs (the largest GPCR family), which expanded to sense diverse stressors like chemicals, hormones, and mechanical cues, integrating them into adaptive responses such as fight-or-flight or metabolic regulation. Thus, bacteriorhodopsin’s role in primordial stress mitigation evolved into the sophisticated GPCR network essential for eukaryotic cellular survival (Figure 1).

Thus, in modern eukaryotes, GPCRs now represent the largest superfamily of membrane-bound proteins that sense and integrate multiple, diverse extracellular signals, including hormones, neurotransmitters, and stress-related molecules like ROS or inflammatory cytokines [3,19,20].

### 3.3. CI and the Sensation of Stress

As stated, these proteins likely evolved as some of the first molecular survival sensors, detecting chemical and physical cues and transducing them via G proteins and various additional adaptors such as β-arrestins [3,21,22], RAMP proteins [23], NHERF [24], and GIT2 [25,26]. These transduced signals then integrate into downstream signaling pathways such as MAPK, PI3K, or cAMP signaling [4,5,19]. This relatively simple view of the flow of cellular information has subsequently been developed and adapted to understand how both short term and long-term cellular functions are also conditioned by intricate networks of GPCR signaling [22,27]. For example, the nuclear factor erythroid 2-related factor 2 (Nrf2) pathway senses oxidative stress by detecting reactive oxygen species (ROS) and activates antioxidant defenses through the transcription of genes like heme oxygenase-1 (HO-1) [8]. Similarly, the DNA damage response (DDR) pathway, orchestrated by proteins like ATM and ATR, detects DNA lesions and coordinates repair or apoptosis [9]. With respect to the role of GPCRs in these systems, the angiotensin II type 1 receptor (AT1R) detects oxidative stress and activates NADPH oxidase, amplifying cellular responses to ROS [28]. Similarly, purinergic GPCRs sense extracellular ATP released during cellular stress, triggering inflammatory or repair pathways [29]. GPCR networks likely complement and interweave other sensory systems including Nrf2, and ATM/ATR kinases. Recent research has also illuminated the role of the relaxin family peptide 3 receptor (RXFP3), in modulating cellular responses to DNA damage and oxidative stress, particularly through its interplay with the scaffold protein GIT2 [30]. RXFP3 expression responds to DNA-damaging agents like camptothecin, while overexpression or stimulation by its ligand, relaxin-3 (RLN3), significantly attenuates damage by reducing phosphorylation of markers such as γH2AX, ATM, and BRCA1, thereby enhancing repair efficiency. This RXFP3-GIT2 axis positions the receptor as a sensor that translocates under stress conditions, integrating DNA damage response (DDR) pathways with cell cycle control to mitigate age-related pathologies, offering promising therapeutic targets for disorders like neurodegeneration and metabolic syndrome [31].

In addition, the angiotensin II type 1 receptor (AT1R) couples with β-arrestins to transduce mechanical (shear/stretch) and oxidative stress signals, promoting adaptive pathways such as ERK (extracellular signal-regulated kinase) activation while modulating NADPH oxidase-derived ROS in vascular cells [32]. β-adrenergic receptors (e.g., β1AR/β2AR) can actively recruit β-arrestins and GRKs under catecholamine or ischemic stress, enabling biased signaling that protects against apoptosis via EGFR transactivation and PI3K/Akt in cardiomyocytes [33]. Stress-responsive receptor systems involving other classes (in addition to rhodopsin-like Class A) of GPCRs have also been documented; for example, the calcium-sensing receptor (CaSR) interacts with filamin A to reorganize actin stress fibers via Rho kinase in response to mechanical or ionic stress, facilitating cytoskeletal adaptations essential for cell survival [34]. Thus, by integrating multiple inputs through shared signaling adaptors, GPCRs enable cells to prioritize and contextualize stressors, forming the sensory backbone of cellular intelligence. Thus, it is evident that GPCR systems allow cells to prioritize dynamic responses to threats and distinguish between transient stressors and long-term repetitive insults that can lead to catastrophic cellular damage. In analogy to GPCR systems, in immune cells, pattern recognition receptors (PRRs) like Toll-like receptors (TLRs) sense pathogen-associated molecular patterns (PAMPs), triggering inflammatory responses [35]. This sensory capacity underscores a cell’s ability to “perceive” its microenvironment with precision, laying the foundation for intelligent decision-making in the future with respect to preparing the range of scope of proteins dedicated to specific response systems. Here, we employ the phrase ‘decision-making’ as a heuristic for the context-dependent selection of signaling pathways to engender various cellular states beneficial to long-term survival.

### 3.4. Systems Integration of GPCR Mechanisms

While GPCRs form a cornerstone of cellular sensory systems due to their diversity and evolutionary conservation, a comprehensive cellular intelligence likely emerges from the coherent integration of multiple overlapping and parallel pathways. For example, receptor tyrosine kinases (RTKs) such as the epidermal growth factor receptor (EGFR) often transactivate with GPCRs to amplify growth factor signals under oxidative stress, while TLRs (Toll-like receptors) in immune cells predominantly sense pathogen-associated molecular patterns (PAMPs) via NF-κB pathways, complementing GPCR-mediated chemokine responses. Nuclear receptors such as peroxisome proliferator-activated receptors (PPARs) mitigate metabolic stress (e.g., lipid imbalances), potentially compensating for GPCR dysfunction in certain contexts. The relative contributions vary by cell type: neurons may rely more on GPCR-RTK crosstalk for synaptic plasticity, whereas innate immune cells prioritize TLR-cytokine receptor integration. Redundancy mechanisms, such as pathway convergence on common effectors (e.g., MAPK or PI3K), ensure systems robustness but can lead to compensation failures in disease states (e.g., TLR overactivation in GPCR-desensitized chronic inflammation). This multifaceted sensory landscape underscores that GPCRs, while central, function within a distributed network

Therefore, such receptor-based signaling networks integrate closely with stress-response pathways to form a rudimentary proto-intelligence framework [8]. The evolution of multicellularity further refined this intelligence, as cells specialized and coordinated via GPCR-mediated intercellular signaling, exemplified by chemokine receptors in immune systems [36] and adrenergic systems for cardiovascular regulation [37]. Epigenetic mechanisms, enabling heritable stress responses, and mitochondrial adaptations, optimizing energy under stress, further enhanced cellular autonomy [6,38]. This evolutionary trajectory suggests cellular intelligence arose from the interplay of early sensory, adaptive, and memory systems, with GPCR networks as a cornerstone, shaped by the need to survive unpredictable stressors.

## 4. Spatial Organization of Cellular Intelligence (CI)

**Figure 2:**
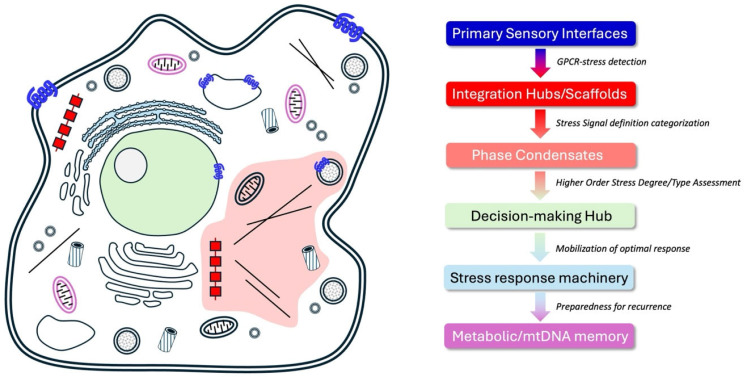
Spatial Subcellular Organization of Cellular Intelligence. The cellular intelligence machinery is created not only of GPCRs but of many interacting cell organelles and phase-restricted structures to aid in the sensation, processing, decision-making and response process deployment. Through an interaction between multiple functional cellular compartments and organelles a profound ‘memory’ of stress can be formed that will persist and protect the cell from future insults. The colors of the text panels are coordinated with the color of the physiological elements in the cell diagram.

Cellular intelligence (CI) mechanisms are likely not confined to a single organelle, phase area of call, or a single protein superstructure, but emerges from distributed molecular signaling networks spanning and interconnecting multiple cellular compartments, signaling domains and endosomic structures (Figure 2). The cell surface plasma membrane, housing a functional sub-population of GPCRs, serves as the primary sensory interface to external stress inputs. Functional interaction with GPCR systems at this site likely generates one of the initial proximal responses to extracellular inputs [3,24,39]. This input, however, involves complex and nuanced interactions with GPCRs that extend to additional signaling systems such as receptor tyrosine kinases, ion channels, proteases and ubiquitinases, that can directly interact with cell surface GPCRs [24,39,40]. The nucleus (and nucleolus) likely acts as a decision-making hub, where GPCR-induced epigenetic modifications, mediated by β-arrestin-scaffolded histone acetyltransferases, regulate gene expression [41]. Mitochondria are likely to be critical for metabolic intelligence, sensing energy stress via AMPK and adjusting mtDNA dynamics in response to these perturbations [10,42]. The endoplasmic reticulum (ER) additionally contributes through stress sensors like IRE1, which activate unfolded protein responses, often modulated by GPCR signaling [43,44]. Cytosolic signaling complexes, including β-arrestin scaffolds, Pyk2 complexes, or GIT2-associated signaling units, integrate these inputs, forming dynamic hubs that coordinate intelligence [21,26,45]. This spatial organization ensures that sensory, adaptive, and memory processes are orchestrated across the cell, with GPCRs as key connectors.

## 5. Emerging Role of Phase Separation in the Spatial Organization of Cellular Intelligence

Cellular decision-making—long viewed as a diffuse, soluble signaling process—now reveals itself as a highly compartmentalized form of subcellular signaling computation. Liquid–liquid phase separation (LLPS) creates membraneless organelles that function as dynamic reaction crucibles, selectively concentrating receptors, adaptors, and effectors into micron-scale hubs with defined material properties [46]. Within these condensates, G protein-coupled receptor (GPCR) signalosomes, centered on β-arrestin scaffolds, transform transient phospho-codes into persistent, reconfigurable states that encode prior stress experience and bias future responses.

For example, it has been shown that β-arrestin itself undergoes phase separation. β-arrestin-1 and -2 contain long intrinsically disordered regions and undergo liquid–liquid phase separation when recruited to activated GPCRs, especially in clathrin-coated pits and endosomal microdomains [47]. In this context, β-arrestin phase separation may provide a physical basis for localizing CI domains, though this concept remains speculative and limited to specific contexts [47]. These β-arrestin condensates may concentrate downstream kinases (JNK3, ERK, AMPK [48]) and scaffold the exact “decision-making hubs” associated with CI. GPCR ‘receptorsomes’ may indeed behave like a phase-separated droplets: it is spherical, undergoes fusion/fission, exchanges components rapidly with the cytosol, and dissolves when phosphorylation or biased ligands shift the interaction valency. Droplet selectively may indeed enrich protective effectors (Nrf2, NF-κB inhibitors) versus destructive ones (JNK3, ASK1) depending on the signaling bias [49,50].

Under prolonged or repeated metabolic/oxidative stress, β-arrestin droplets fuse with or seed stress granules (G3BP1/TIA-1 positive). These granules store mRNAs of adaptive response genes and literally constitute a phospho-epigenetic memory that is read out hours to days later [51,52]. In neurons, disrupting LLPS of these granules accelerates amyloid and tau pathology and erases long-term potentiation [53,54]. Thus, far from being passive crowding phenomena, these phase-separated droplets may actively shape cellular intelligence by (i) establishing privileged microenvironments where weak, multivalent interactions among intrinsically disordered regions of GRK and β-arrestin proteins drive selective effector recruitment, (ii) enabling rapid switching between fluid exploratory states and gel-like memory states, and (iii) providing a physical basis for threat prioritization through tunable partition coefficients and droplet surface tension.

Phase-separated condensates themselves exhibit fractal-like clustering in space and power-law size distributions in time [55,56]. Chronic disease states (Alzheimer’s, Parkinson’s, cancer) systematically reduce the fractal dimension of these condensates and increase their gelation/solidification—this could represent a physical manifestation of the “loss of adaptive memory” scales up from single cells eventually to dementia. Subcellular phase separation may indeed represent the main physical mechanism that turns a diffuse cloud of adaptor proteins into discrete, reconfigurable “computing droplets” that store experience, integrate multimodal threats, and execute biased responses. Recognizing phase separation as the structural foundation of cellular intelligence shifts the paradigm from linear pathways to liquid-state computing: intelligence is not merely executed within the cell—it is physically sculpted by the reversible condensation of its core decision-making machinery. This perspective not only explains the remarkable context-sensitivity of GPCR signaling but also identifies condensate material properties as a new and druggable dimension of cellular cognition.

## 6. Reactive and Adaptive Responses: Decision-Making Under Stress

**Figure 3:**
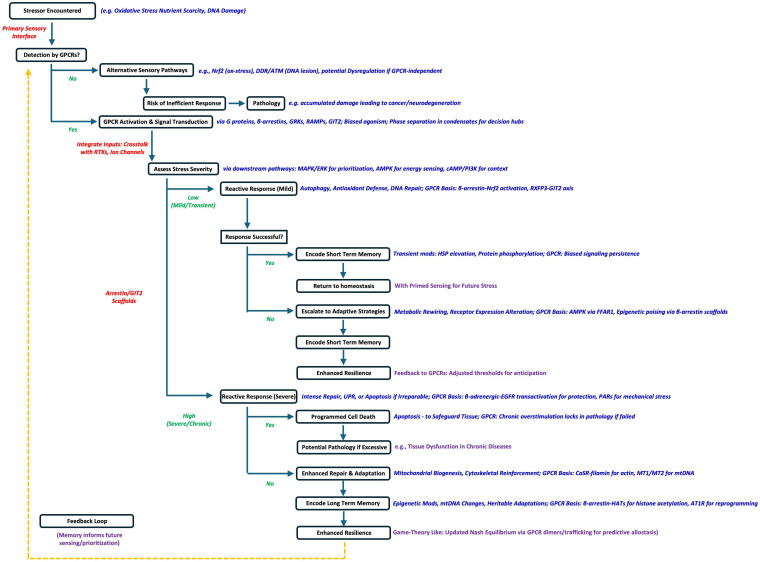
GPCR-based Stress Sensory Routine. In response to a stochastic perturbation, the cell enters into a decision-making tree that assesses the stress and measures the appropriate response to the stress to ensure continued survival and optimal health and longevity. Successful information journeys across this decision tree will allow for a more rapid and reliable mitigation process to stress-related cellular insults.

Upon detecting stressors, cells need to engage in a game-like process where they select the ideal deployment of immediate reactive responses to most successfully mitigate the potential cellular damage [20]. In the context of long-term cell survival, the ‘game’ here is to maximize the likelihood for continued cellular function; hence, damage has to be minimized, metabolic energy has to be conserved with respect to dynamic stress responses, and protein deployment and microcomplex generation need to be judicial and potentially predictive of future stressful inputs. Thus, the initial response(s) to stress are followed by optimized adaptive strategies to enhance long-term resilience and preserve energetic or proteostatic loss following the stress. For a cell to maximize its capacity to effectively avoid catastrophic damage that could induce long term injury, the most efficient and rapid response and mitigation programs must be enacted to (i) attenuate damage, (ii) encourage rapid repair and cellular realignment, and (iii) simultaneously gauge the efficiency and qualitative nature of the response to enable the creation of a stress memory. The molecular integration of these cellular responses—in a vertical and horizontal organizational manner—thus aims to produce the least amount of excessive ‘response stress’ caused by undue transcriptomic and/or proteomic responses. In this general cellular context, reactive responses can include GPCR signaling-controlled rapid mechanisms like DNA repair [30], autophagy, or programmed cell death [57,58]. For instance, base excision repair (BER) corrects oxidative DNA lesions, while autophagy clears damaged mitochondria to prevent ROS amplification [6,59]. In extreme cases, apoptosis eliminates irreparably damaged cells, safeguarding tissue integrity [60]. Cellular adaptive responses, however, reflect a deeper layer of intelligence, as cells reconfigure their molecular signaling architecture to withstand future insults in the most energy- and resource-efficient manner. For example, under glucose deprivation, cells shift from glycolysis to fatty acid oxidation, a metabolic adaptation mediated by AMP-activated protein kinase (AMPK) [10]. In neurons, repeated oxidative stress induces the expression of neuroprotective genes, enhancing resilience to ischemia [61,62]. Epigenetic modifications, such as histone acetylation or DNA methylation, further enable cells to “lock in” adaptive states, altering gene expression profiles to match environmental demands [11]. These responses suggest a form of cellular decision-making, where immediate survival is balanced against long-term stability. This mechanistic process can be considered as a simple program that is executed in the cell each time a stochastic stress event occurs (Figure 3). With each successive stress, for a given cell’s survival, this sensation and response program will be refined and optimized in an agile and dynamic manner.

## 7. Short- and Long-Term Memory Processes: Learning from Stress

The notion of cellular memory—both short-term and long-term—is perhaps the most provocative aspect of the CI hypothesis. Short-term cellular memory may manifest itself as transient molecular changes that prime cells for recurring stressors. For example, heat shock proteins (HSPs) remain elevated in their expression after thermal stress, conferring temporary resistance to subsequent heat exposure [62,63]. Similarly, in immune cells, trained immunity involves transient epigenetic and metabolic reprogramming that enhances responses to secondary infections [12]. Crucially, GPCR signaling systems play a pivotal role in these processes, acting as sensors that detect stressors and initiate cascades leading to memory formation. For instance, in the model organism C. elegans, the GPCR SRZ-75 in sensory neurons coordinates a systemic mitochondrial unfolded protein response (UPRmt) via Gαq signaling, enabling cells to “remember” mitochondrial stress and mount faster, more robust defenses against future oxidative or metabolic insults [64]. This GPCR-driven mechanism illustrates how environmental cues are transduced into cellular adaptations, potentially persisting through epigenetic marks on nuclear or mitochondrial DNA (mtDNA).

Long-term memory, in contrast, typically involves stable or heritable changes that encode stress history. The molecular mechanisms that support long-term stress memory need a stable structural profile to maintain the signaling encryption of the stress while still being flexible enough to be de-encrypted by the CI system to ‘replay’ the experience of the initial cell stress and to organize long-term adaptive responses to this stress. Maintaining the quality of encryption of the memory data may serve as a vital aspect of translating rapid stress responses into longer term homeostatic cellular resets. To this end, epigenetic modifications, such as DNA methylation of stress-response genes, can persist across cell divisions, as seen in cancer cells that develop drug resistance after repeated chemotherapeutic exposure [13]. Mitochondrial adaptations also contribute to long-term memory; for instance, chronic oxidative stress can alter mitochondrial DNA (mtDNA) copy number or function, shaping cellular metabolism for generations [42]. GPCRs further enhance this by directly or indirectly modulating mitochondrial and epigenetic landscapes. Melatonin receptors (MT1/MT2) localized on mitochondrial membranes sense oxidative stress, regulate melatonin synthesis within mitochondria, and trigger signaling that protects against ROS-induced damage, potentially imprinting long-term changes in mtDNA heteroplasmy or epigenetic regulators like SIRT1 for sustained cellular resilience [65]. Similarly, the angiotensin II type 1 receptor (AT1R), a GPCR, integrates oxidative and mechanical stress through Gα13 coupling, influencing mitochondrial dynamics and epigenetic reprogramming in vascular cells to foster adaptive memory against chronic hypertension or inflammation [66]. In bacteria, CRISPR-Cas systems provide a striking analogy, storing genetic “memories” of viral infections to guide future defenses [67]. While eukaryotic cells lack such explicit systems, their ability to retain stress-induced changes—often orchestrated by GPCR networks—suggests a memory-like capacity, enabling anticipation and preparation for future challenges. These GPCR-involved pathways, whether through mitochondrial modifications or DNA methylation patterns, convincingly demonstrate how cells “learn” from stress, deploying pre-encoded responses to enhance survival in fluctuating environments.

## 8. Implications for Long-Term Disease: When Intelligence Fails

**Figure 4:**
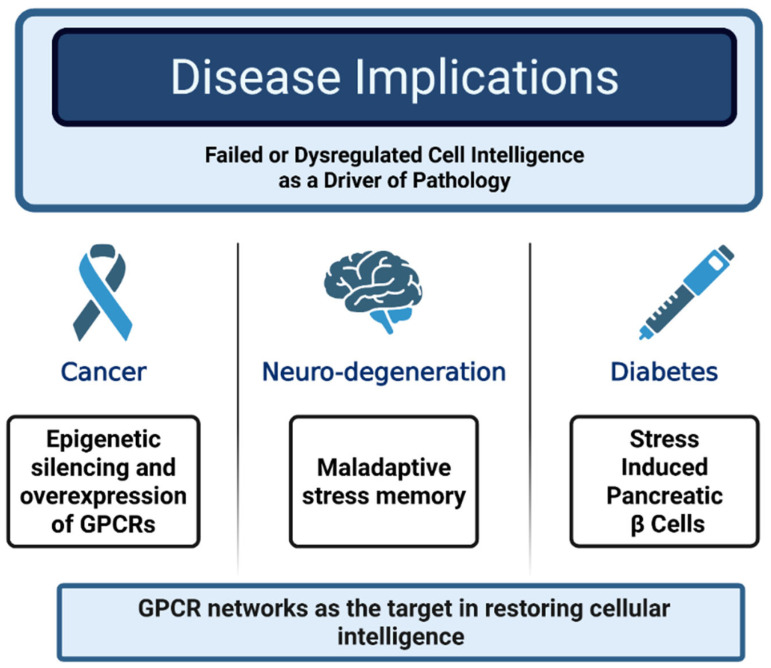
Disease Implications of Dysregulated Cellular Intelligence. An inability to efficiently sense, assess, react and respond to stochastic stress renders cells frail and more susceptible to further damage, leading them into a cycle of augmented disease progression each time a stress cycle is poorly managed.

The cumulative effects of the dysregulation of cellular intelligence and stress memory formation likely plays a critical role in the pathogenesis of chronic diseases. Failures in sensory mechanisms, such as diminished DDR in aging cells, allow stressors like DNA damage to accumulate, increasing cancer risk [1]. Maladaptive responses, such as chronic inflammation driven by overactive immune cells, promote tissue dysfunction in diseases like atherosclerosis or Alzheimer’s [2]. Dysregulated cellular memory, such as aberrant epigenetic silencing of tumor suppressor genes, can lock cells into pathological states, as observed in cancer or fibrosis [14]. For example, in type 2 diabetes, pancreatic beta cells exposed to chronic hyperglycemia develop epigenetic changes that impair insulin secretion, perpetuating metabolic dysfunction [68]. Similarly, in neurodegeneration, neuronal “memory” of oxidative stress may drive progressive protein misfolding, as seen in Parkinson’s disease [69]. These examples highlight how cellular intelligence, when misdirected, translates daily stressors into long-term disease, underscoring the need to target these processes therapeutically. Spanning perception, response, adaptation, and memory, when cellular intelligence fails or is dysregulated, cells can lock into maladaptive states. The dysfunction of cellular intelligence contributes to chronic diseases such as cancer (via epigenetic silencing and overexpression of GPCRs), neurodegeneration (via maladaptive stress memory), and diabetes (via β-cell dysfunction). Understanding these positions of the intelligence breakdown highlights potential in therapeutics that target restoring intelligent cellular responses (Figure 4).

## 9. How GPCRs Generate and Mediate the Phenomenon of Cell Intelligence

We hypothesize that cells—both prokaryote and eukaryote—function as adaptive entities that perceive, integrate, and respond to stressors in a manner analogous to decision-making systems, supported by evidence from GPCR dynamics [16,20,62]. Cells therefore can be considered—at some level—to be cognitively active, decision-making entities that continuously perceive, interpret, decide, act, and—crucially—remember. In the face of relentless stochastic attack (oxidative stress, proteotoxic insults, DNA lesions, nutrient fluctuation, inflammatory signals, and environmental toxins), survival demands far more than blind homeostasis. Instead, the relentless attack of aging requires genuine intelligence at the single-cell level: the capacity to detect threats with exquisite specificity, integrate multimodal inputs across space and time, mount proportionate and context-appropriate responses, and encode memory of past encounters to pre-emptively re-challenge. At the very center of this cell-autonomous intelligence stand GPCR signaling networks—an extraordinarily dense, versatile, and evolvable sensory apparatus that functions as the cell’s “nervous system.” GPCRs do not merely receive individual ligand information in isolation; they form vast, highly interconnected super-networks linked by shared heterotrimeric G proteins, β-arrestins, GRKs, RGS proteins, and downstream effectors [4,70,71,72]. This architectural convergence generates both vertical signal integration (from plasma membrane to nucleus) and horizontal crosstalk (receptor–receptor, pathway–pathway, and even organelle–organelle), creating a distributed computational mesh capable of non-linear, emergent cell resilience processing. Through biased agonism, allosteric modulation, receptor heterodimerization, transactivation of tyrosine kinase receptors, and scaffolded compartmentalization, GPCR ensembles achieve something remarkable: they transform a chaotic barrage of extracellular and intracellular stressors into coherent, prioritized perceptual representations [19,24,39,73,74]. Oxidative stress sensed via angiotensin AT1 or adrenergic receptors, DNA damage signals relayed through protease-activated receptors (PARs) or frizzled, metabolic stress detected by free fatty acid or succinate receptors all converge onto common nodes (PKA, PKC, MAPK cascades, AMPK, Ca^2+^ waves, Rho GTPases, β-arrestin hubs) that function as molecular “logic gates” for threat assessment [8,9]. The effector layer for dynamic coordinated stress responses is equally sophisticated: (i) acute survival: rapid induction of autophagy, UPR, antioxidant responses, and DNA repair [10]; (ii) adaptive reprogramming: metabolic rewiring, mitochondrial biogenesis, cytoskeletal fortification, and immune-like secretion of damage-associated molecular patterns (DAMPs; [75]); (iii) long-term anticipatory memory: GPCR-driven chromatin remodeling, DNA methylation, histone modification, and even non-genetic inheritance via trained immunity-like mechanisms [11,12,13]. Crucially, these networks exhibit bona fide memory and learning. Repeated or primed GPCR activation induces prolonged β-arrestin signaling, receptor desensitization/resensitization cycles, and epigenetic poising that can persist for hours, to generations. This is cellular “experience” encoded in molecular form—cells literally remember oxidative bursts, inflammatory episodes, or nutrient deprivation and adjust future response thresholds accordingly. When this GPCR-centric intelligence falters—through mutation, chronic ligand overstimulation, or loss of adaptor fidelity—the cell loses its ability to correctly perceive or prioritize threats. The result is pathological hyper- or hypo-reactivity: cancer (sustained proliferation and survival signaling), neurodegeneration (failed proteostasis and chronic inflammation), metabolic syndrome, and autoimmunity [14]. Far from being mere druggable switches, GPCRs and their extended interactomes constitute the closest thing biology has to a cellular brain: a highly plastic, self-organizing sensory–motor–memory system that endows even unicellular organisms with adaptive intelligence. Recognizing this shifts the paradigm from “cells react” to “cells decide, learn, and anticipate.” The therapeutic implications are profound: instead of targeting single receptors, we can now contemplate reprogramming entire GPCR intelligence networks—restoring perceptual accuracy, recalibrating decision thresholds, and even installing synthetic memory—to treat the root cause of chronic disease: the loss of cellular wisdom.

## 10. GPCR Networks as Sensory Hubs of Cellular Intelligence

An individual cell is not as a mere biochemical automaton, blindly churning through metabolic pathways and kinase activation steps. It is, however, more akin to an ever-vigilant oracle for the state of healthy cellular homeostasis. The sensory core of these systems is the GPCR with its associated coterie of signaling adaptors [22]. These adaptors are nuanced intermediaries that contend with the extreme sensory chaos into the refined output of adaptive intelligence and stress resilience.

### 10.1. β-Arrestin Dynamics

Our proposed GPCR network supports what could be thought of as a learning entity that measures and evaluates the probability of cell survival in any given stress state, integrating metabolic challenges, oxidative species attack, altered proteostasis and the potential to end up in apoptotic or senescent states [7,76,77]. For example, β-arrestin has been shown, through various structural analyses, to be a complex controller of highly nuanced GPCR signaling [78,79]. GRK2-mediated phosphorylation of the angiotensin II type 1 receptor (AT1R) engenders a rapid interaction with β-arrestin-2 simultaneously attenuating Gq-mediated calcium mobilization, scaffold-based control of ERK1/2 cascades, and eventual translocation to the nucleus to reprogram gene expression [80]. This scenario represents a highly complex desensitization process where β-arrestin-2’s “finger loop” and “β-strand insertion” motifs dock with phosphorylated pseudosites, dictating whether the signal diminishes or rises into adaptive resilience [81]. β-arrestin association with GPCRs is also functionally conditioned via phosphatidylinositol 4,5-bisphosphate (PI(4,5)P2) in the membrane where it biases β-arrestin engagement, favoring tail versus core engagement and thus tilting the scales toward pro-survival autophagy or deleterious unchecked proliferation [80].

### 10.2. GRK Encoded Protein Phosphorylation

Selective GRK-mediated phosphorylation of receptors also occurs in a manner that can also help encrypt cellular resilience signals. This phosphorylation process has been shown to regulate cross talk from canonical to non-canonical signaling pathways and promote differential sensitivities to diverse input signals. Hence, GRK6 phosphorylation (assisted by recruitment via the COMMD3/8 scaffold) of CXCR4 engenders of phosphspecific imprint that attracts β-arrestin-1 to activate PI3K/Akt, directing B-cell chemotaxis [82,83]. GRK phosphorylation clusters (multisite barcodes on receptor C-termini) dictate the ultimate β-arrestin conformation with the receptor, from the “partial engagement” mode to the “full tail” clasp that sustains endosomal trafficking which can result in the imprinting of immunological memory against recurrent assaults [84,85].

### 10.3. GPCR Sensory Hubs in Neurometabolic Diseases

If this sensory system is disrupted, e.g., caused by chronic inflammation induced GRK2 hyperactivity, resultant rheumatoid autoimmunity may occur, while reductions in GRK2 activity can engender unchecked cytokine storms [86]. In neuronal systems, metabotropic glutamate receptors (mGluRs) undergo GRK4/5-mediated phosphorylation, which recruits β-arrestin-2. This interaction scaffolds JNK signaling and supports synaptic plasticity while countering amyloid-β toxicity in Alzheimer’s disease models [87,88]. mGluR5 engages β-arrestin to influence tau kinase activation. Biased signaling, induced by allosteric modulators, shifts downstream signaling toward neuroprotective outcomes, including NF-κB inhibition, reduced microglial activation, and preservation of dendritic structures [89,90]. In Parkinson’s disease, excessive GRK2 phosphorylation of dopamine D2 receptors reduces β-arrestin recruitment to ERK pathways. This disrupts dopaminergic signaling and promotes α-synuclein aggregation [91]. β-arrestin links membrane signaling to mitochondrial and broader cellular stress responses. It integrates inputs from receptor tyrosine kinases (RTKs) and integrins, allowing detection of glutamate surges alongside mechanical stress or metabolic/vascular deficits [92,93]. Hence, GPCR networks can function as distributed systems, with biased agonism—now measurable via advanced BRET-FRET techniques—enabling prioritized responses to threats [94]. For example, upon FFAR1 sensation of metabolic stress, it is phosphorylated by GRK3 phosphorylation, which induces β-arrestin scaffolding of AMPK for the regulation of energy homeostasis [95]. Oxidative stress sensation via the AT1R causes a β-arrestin-2-dependent recruitment of Nrf2 to mount an anti-oxidative response [96]. PAR2 receptors have been shown to respond to mechanical stress cues, and in response to this, it is phosphorylated by GRK5 phosphorylation, which subsequently mediates a β-arrestin-dependent activation of RhoA for cytoskeletal reinforcement [73,85].

These mechanisms represent adaptive cellular responses, encoding prior insults through phosphorylation patterns to guide future adaptations. Dysregulation of this sensory system unsurprisingly contributes to pathology, e.g., β-arrestin biases promote metastasis in cancer or tau pathology in neurodegeneration [14,97]. Therapeutic strategies involving cryo-EM structural insights of β-arrestin-GPCR interactions and AI-driven design of ligands that can recalibrate adaptor interactions, restoring balanced signaling to the sensory system represent important rationale points of future drug discovery to augment the GPCR sensory apparatus [98,99].

## 11. GPCRs as Game-Theory Strategists: Cellular Nash Equilibria in the Repeated Stress Game

Every stress encounter is not a one-off crisis; it is a single move in an infinite repeated game against an unpredictable, damage/stress causing opponent. The cell may act as a rational, yet opportunistic, player whose goal is simple: survive now, reproduce later, minimize energy expenditure, and maximize future flexibility. In this evolutionary survival game, GPCR networks function as the responsive and strategic unit of the cell—they detect the opponent’s move (stressor), recall payoff/recovery histories from previous challenges, compute expected depletions to utilities, and then deliberately shift the expression, trafficking, dimerization, and biased-signaling profiles of hundreds of GPCRs to converge on a new Nash equilibrium that is optimally adapted to the anticipated next insult wave [100,101,102,103]. In this paradigm, the cell does not merely react; it learns. Each new exposure to stress generates a payoff vector (damage incurred, ATP spent, proteostasis cost, epigenetic drift) that is stored as durable molecular memory in the form of altered GPCR phospho-barcodes, β-arrestin recruitment thresholds, GRK expression levels, receptor heterodimer repertoires, and even locus-specific chromatin poising [104,105,106]. Subsequent encounters are met with a revised strategy: receptors that previously yielded suboptimal payoffs are downregulated or trafficked to lysosomes, while winning receptor ‘gambits’ are transcriptionally amplified, lipid-raft enriched, or forced into novel dimers that unlock higher-utility pathways. These activities are supported by evidence acquired from stress response analyses from multiple research groups. After repeated hypoxic episodes, endothelial cells learn to favor the S1P1–AT1R heterodimer over monomeric AT1R signaling. This heterodimer biases toward engaging the β-arrestin-2-Nrf2 antioxidant defense strategy instead of the Gq-ROS production, output dramatically raising survival probability in the next round of stress exposure [107,108]. In addition, dopaminergic neurons exposed to intermittent oxidative attack upregulate DRD2–GRK6 complexes that scaffold and promote the AKT–GSK3β axis, while simultaneously suppressing pro-apoptotic JNK signaling. The same stressor applied naïvely triggers cell death after the first round; after three rounds, the neuron has “learned” to defend itself against death and cooperate with survival, with payoff curves shifting by >40% [106,109]. As a further example, chronic low-grade FFA exposure to pancreatic β-cells induces a losing strategy of Gq-driven insulin hypersecretion and ER stress. This deleterious response, however, is replaced, after several cycles, by a preferential FFAR1–GRK2–β-arrestin-1 signaling paradigm that activates AMPK and mitochondrial biogenesis while desensitizing the Gq arm. Here, the cell has executed a minimax strategy: minimize the maximum future damage [110,111]. These are not prosaic metaphors to convey a higher-order concept; they are experimentally observable shifts in receptor expression and signaling bias that directly correlate with previous stress history and future resilience [112,113]. The machinery of this game-theoretic adaptation is exquisitely GPCR-centric: Payoff evaluation is engendered through β-arrestin and GRK isoforms acting as reckoning factors, integrating duration, amplitude, and compartmentalization of signaling to compute “net utility” of the attack–response matrix [114]. For memory storage, persistent phospho-barcodes and epigenetic acute modification responses linked to GPCR loci (e.g., H3K27ac enrichment at ADORA2A after repeated adenosine surges) encode the updated payoff matrix [115,116]. To enable the scope for strategy revision, pioneer transcription factors (CREB, SRF, MEF2) driven by sustained β-arrestin-ERK nuclear signaling rewrite the GPCR transcriptome, selectively amplifying receptors with proven to yield higher future expected payoffs [117]. For the execution of new resilience equilibrium there are induced alterations to receptor trafficking, dimerization, and allosteric biases that generate a new signaling repertoire that is no longer naïve but engineered for repeated stress responses [62,103,118]. In essence, GPCR networks transform the cell into a Bayesian strategist that continuously updates its prior (naïve receptor landscape) into a posterior (stress-optimized landscape) using stress history as direct evidence. The result is not un-guided homeostasis but predictive allostasis: the cell does not merely return to baseline; it moves to a new, superior baseline calibrated to the statistical structure of its own personal threat environment. This is cellular intelligence stripped to its evolutionary core: a self-organizing, game-theoretic learning system whose aim is survival probability.

We have proposed this novel game-theoretic framework as a heuristic model to conceptualize GPCR-mediated adaptations, drawing analogies from evolutionary game theory. While supported by experimental observations of history-dependent signaling biases [107,109], firm and direct mathematical formalization or comprehensive validation at the cellular level will be eventually required to establish this concept in a concrete manner. Hence, future studies could test predictions, such as quantifying ‘payoff matrices’ through single-cell signaling dynamics under iterative stressors.”

## 12. GPCR Networks in Cellular Memory Formation

GPCR networks, via common signaling adaptors, are critical for establishing cellular memory, enabling cells to “learn” from stress. Short-term memory is exemplified by GPCR-mediated transient signaling. For instance, differential arrestin-subtype-mediated histamine H1 and oxytocin receptor signaling in the myometrium can be used to encode differential forms of muscular activity around the regulation of functional actions during labor [119]. In immune cells, C5a receptors drive trained immunity by recruiting β-arrestin to promote histone methylation, enhancing cytokine production upon re-exposure to pathogens [12,120]. Long-term memory relies on GPCR-induced epigenetic and mitochondrial changes. The serotonin 5-HT2A receptor, for example, recruits β-arrestin to scaffold DNA methyltransferases, silencing stress-response genes in neurons, a mechanism implicated in psychiatric disorders [121]. In metabolic cells, free fatty acid receptor 4 (FFAR4) signaling alters mtDNA dynamics via cAMP, encoding oxidative stress history [122]. By modulating epigenetic landscapes and organelle function through shared adaptors, GPCR networks embed stress memories, allowing cells to anticipate and adapt to future challenges. GPCRs have been shown to form a type of memory formation, e.g., C5a receptors promote trained immunity via β-arrestin-mediated histone methylation [120]. Serotonin 5-HT2A receptors silence genes via DNA methylation, impacting psychiatric disorders [121]. Free fatty acid receptor 4 (FFAR4) alters mtDNA, encoding stress history [122].

Cellular intelligence operates through organelles, molecular activities, and feedback loops. Key organelles include mitochondria, which sense energy stress and signal via ROS or AMPK [6]; the ER, which manages protein folding stress [43]; and the nucleus, which encodes memory via epigenetic marks [11]. Molecular activities include GPCR signaling, metabolic enzyme activation, and transcription factor modulation (e.g., Nrf2) [8]. Feedback loops are critical: negative feedback via GRKs desensitizes GPCRs, maintaining homeostasis [21], while positive feedback, like ROS-induced Nrf2 activation, amplifies responses [28]. GPCR networks integrate these components, as seen in GLP-1R-driven AMPK-mitochondrial feedback, which optimizes energy under stress [123]. This interplay forms the mechanistic core of cellular intelligence.

## 13. GPCR Signaling and Epigenetics in Cellular Stress and CI

Cellular intelligence enables cells to sense environmental stressors, execute context-specific responses, adapt, and retain molecular memories to anticipate future challenges, underpinning resilience and health. Dysregulation contributes to chronic diseases like cancer, neurodegeneration, and metabolic disorders. G protein-coupled receptors (GPCRs) serve as sensory interfaces, while epigenetic modifications encode stress memory. This review examines their interplay, disease implications, and therapeutic potential. GPCRs detect stressors with precision. Reactive oxygen species (ROS) modulate angiotensin II receptors (AT1R), activating MAPK pathways for cell survival [124]. GPR120 senses fatty acids, engaging PI3K/AKT for metabolic homeostasis [125], while CXCR4 detects cytokines, promoting migration or survival in inflammation [126]. These responses mitigate acute damage and interface with epigenetic machinery for long-term adaptation. Epigenetic modifications—DNA methylation, histone modifications (e.g., acetylation, methylation), and non-coding RNAs (e.g., miRNAs, lncRNAs)—encode stress history without altering DNA sequence [127,128,129]. Acute stressors like heat shock induce histone acetylation at genes like HSP70 for protection [130]. Chronic stress causes stable DNA methylation, as in hypermethylated tumor suppressor genes in cancer [131]. Histone modifications in neurons enhance resilience to neurotoxic insults [132], enabling transient and heritable stress memory.

**Figure 5:**
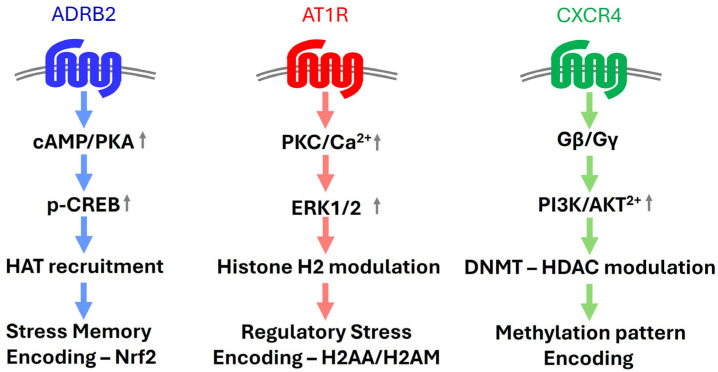
GPCR signaling and epigenetic modulation mediates the capacity for stress encoding. GPCR activation, like β-adrenergic receptors, increases cAMP, activating PKA and CREB, which recruits HATs like CBP/p300 for histone acetylation at genes like NRF2. AT1R activates MAPK/ERK, causing complex histone H2 modification patterns for gene expression/chromatin structure regulation. CXCR4-driven PI3K/AKT modulates DNMTs and HDACs, altering methylation patterns. Grey arrows indicate increases in activity of the protein or kinase.

GPCR signaling and epigenetics form feedback loops (Figure 5). GPCR activation, like β-adrenergic receptors, increases cAMP, activating PKA and CREB, which recruits HATs like CBP/p300 for histone acetylation at genes like NRF2 [133]. AT1R activates MAPK/ERK, phosphorylating histone H3 for immediate–early gene expression [134]. CXCR4-driven PI3K/AKT modulates DNMTs and HDACs, altering methylation patterns [135]. Conversely, epigenetic marks regulate GPCR expression: DNA methylation silences GPR120 in metabolic disorders [136], histone acetylation upregulates CXCR4 in inflammation [137], and miR-21 targets GPCR mRNAs [129]. This loop enables cells to sense, react, adapt, and remember stressors [131,132,138]. Dysregulation of this GPCR-epigenetic axis drives disease. In cancer, CXCR4 signaling induces hypermethylation of tumor suppressor genes, promoting metastasis [139]. In neurodegeneration, defective histone acetylation in Alzheimer’s exacerbates neuronal loss [140]. Therapeutically, modulating GPCRs with β-blockers or chemokine antagonists could prevent maladaptive epigenetic changes [5]. HDAC inhibitors (e.g., vorinostat) or DNMT inhibitors (e.g., azacitidine) may reverse aberrant gene expression [141]. Single-cell omics could map GPCR-epigenetic interactions for personalized therapies [142]. Systems biology and single-cell epigenomics could model these networks, uncover cell-specific stress memory, and explore non-coding RNAs as mediators, revealing new targets to enhance resilience or reverse pathology. The GPCR-epigenetic axis drives cellular intelligence, with GPCRs sensing stressors and epigenetics encoding memory. Dysregulation causes disease, but targeting this axis offers therapeutic promise. Advances in systems biology and omics will deepen insights, paving the way for innovative treatments.

## 14. Future Directions

In this review, we have assessed the current evidence linking coordinated GPCR signaling networks—woven together using stress-sensitive receptor adaptors—that facilitate a capacity for cells to be sensitive to stochastic stressors. This ability of GPCR-supported sensation, perception, acknowledgement, responsivity and memory is analogous to primitive levels of ‘intelligence’. The evidence collected here suggests that disruptive changes to the stress–sensory GPCR networks are associated with the etiology of chronic disease, including cancer, heart failure, tissue fibrosis, neurodegeneration and T2DM [1,14,42,68,139,140,143].

### 14.1. GPCR Adaptor Dysfunction

Disease-associated GPCR network failure is potentially driven through alterations in the balance of adaptor protein functions [7]. In cancer, overexpressed GPCRs such as CXCR4 enhance tumor survival by recruiting β-arrestin to activate oncogenic MAPK pathways, promoting metastasis [144]. In neurodegeneration, dysregulated mGluR signaling disrupts β-arrestin-mediated neuroprotection, accelerating protein misfolding in Alzheimer’s disease [145]. In metabolic disorders, GLP-1R desensitization due to GRK overexpression impairs adaptive mitochondrial responses, exacerbating insulin resistance [123]. Chronic inflammation, a hallmark of many diseases, is fueled by dysregulated chemokine GPCR signaling, where β-arrestin biases toward pro-inflammatory pathways [36]. These examples illustrate how GPCR network dysregulation—through altered adaptor interactions—disrupts cellular intelligence, transforming adaptive responses into pathological drivers. Targeting GPCR-adaptor interactions, such as with biased agonists that selectively modulate β-arrestin signaling, offers a promising therapeutic strategy [146].

### 14.2. Exploiting Cellular Intelligence and GPCR Networks

The centrality of GPCR networks in cellular intelligence raises critical questions. How do adaptors prioritize signaling outputs under complex stress? What distinguishes adaptive from maladaptive GPCR-mediated memory? Can we modulate GPCR networks to prevent disease? Single-cell omics can reveal heterogeneity in GPCR signaling, uncovering cell-specific responses to stress [147]. Systems biology models can map GPCR–adaptor interactions, identifying therapeutic targets [148]. CRISPR-based tools may enable precise editing of GPCR-induced epigenetic marks, resetting pathological memory [149]. Pharmacologically, biased GPCR agonists or allosteric modulators targeting adaptor interactions hold promise for fine-tuning cellular intelligence [146,150]. Recent advances in single-cell omics and systems biology have begun to unravel these mechanisms, revealing strategies to prevent or reverse stress-induced pathologies [142,148].

Future research should prioritize dissecting GPCR network dynamics to harness cellular intelligence for therapeutic benefit. High-resolution techniques, like single-molecule imaging, can elucidate adaptor–GPCR interactions in real time, revealing how β-arrestins or GRKs shape signaling specificity [151]. Computational modeling of GPCR networks can predict how adaptor-mediated crosstalk influences stress responses, guiding drug design [152]. Therapeutically, modulating GPCR networks offers exciting prospects. For example, biased agonists of AT1R that favor β-arrestin over G protein signaling reduce oxidative stress in hypertension without adverse effects [153]. In cancer, antagonists of CXCR4 can disrupt β-arrestin-driven metastasis, restoring cellular homeostasis [144]. In metabolic diseases, GLP-1R agonists enhance adaptive responses by amplifying cAMP signaling, improving insulin sensitivity [154]. By targeting GPCR–adaptor interactions, we can recalibrate cellular intelligence, preventing or reversing stress-induced pathologies.

### 14.3. Cellular Fractals of Disease

**Figure 6:**
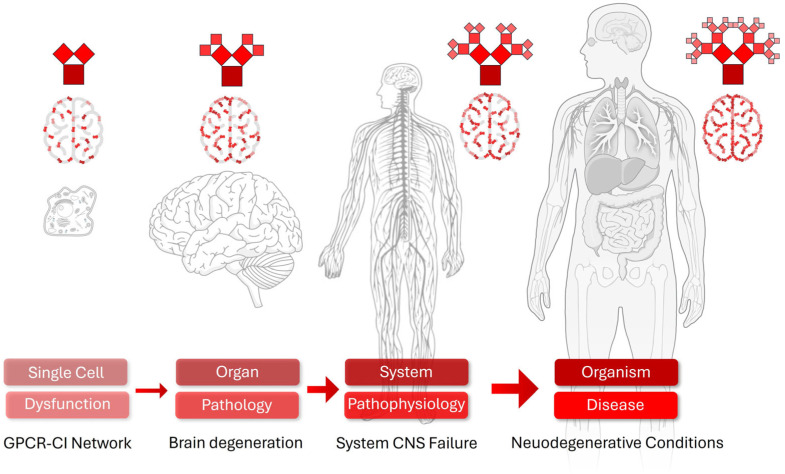
The fractal-like structure of disease based on GPCR stress response systems. Organism-level expression of disease can be connected back down to the single cell level through the lens of the GPCR stress sensory and response network. In this example, dysfunction of the GPCR cellular intelligence network in single brain cells grows and develops (in a similar way to the growth of the represented red Pythagorean fractals) into organism level disorder, e.g., Alzheimer’s disease. Thus, the disease represents simply a more elaborate version of the initial single cell dysfunction of the GPCR-generated cellular intelligence entity.

So, if there is a core function of cells to possess CI, and cellular dysfunction may be a downstream sequalae of this, then perhaps it is possible for whole somatic disorders, e.g., Alzheimer’s disease, T2DM, and CKD, to be observed in an individual all the way down from the gross organ system level to the individual cell level in a functionally analogous manner. Hence, dementia may be associated with an observable and testable loss of CI, while T2DM could be similarly displayed at the level of energy management systems during cell stress. This concept of disease as a fractal cascade—from disrupted cellular stress memory to organismal cognitive decline—finds substantial support in systems biology and fractal physiology literature. Fractals refer to self-similar patterns across scales, as observed in biological systems [155]. Fractal geometry, characterized by self-similar patterns across scales, permeates biological organization, from mitochondrial networks to neural architectures and whole-brain dynamics. Disruptions in stress-response mechanisms at the cellular level, such as impaired phospho-epigenetic encoding of prior insults via β-arrestin scaffolds, mirror hierarchical failures in tissue homeostasis and organismal adaptation. In neurodegeneration, for instance, chronic cellular stress elevates cortisol, eroding hippocampal neurogenesis and synaptic plasticity—key substrates for memory consolidation—while fractal analyses reveal reduced dimensional complexity in neuronal morphologies and cortical ribbons, signaling progression to dementia [155,156,157]. These patterns extend beyond the brain: elevated glucocorticoid signaling impairs astrocytic support networks, fostering neuroinflammation that scales up to gliosis and vascular dysregulation, akin to the self-propagating aggregates seen in amyloid-β or α-synuclein pathologies [158,159] (Figure 6). Longitudinal cohorts further substantiate this, showing that midlife chronic stress doubles dementia risk through additive effects on mild cognitive impairment trajectories, with PTSD cohorts exhibiting twofold higher incidence via HPA-axis hyperactivity [160]. Hence, GPCR desensitization in single neurons may propagate via inflammatory DAMPs, amplifying gliosis at the tissue level and contributing to hippocampal atrophy observed in dementia cohorts [158,160].

If biological systems embody scale-invariant resilience, then disease fractals represent a breakdown in this distributed intelligence, where local cellular “forgetfulness”—failure to adaptively encode stress via GPCR hubs—propagates to global perceptual deficits. This is not mere analogy but mechanistic continuity: just as biased agonism recalibrates mitochondrial ROS quenching in single neurons, organismal stress memory falters when vascular or immune feedback amplifying the signal, tipping attractors toward pathological steady states [161,162]. In cancer, for example, fractal elevations in tumor cell proliferation dimensions correlate with metastatic invasion, paralleling how amyloid fractals in Alzheimer’s erode dendritic filigrees into neurofibrillary tangles [163,164]. Such universality suggests a provocative paradigm: diseases are not isolated failures but echoes of systemic decoherence, where GPCR-derived cellular memory lapses fractalize into organ dysfunction and behavioral entropy. Embracing this view demands a shift from siloed interventions to multiscale recalibration—leveraging AI-optimized allosteric modulators to restore subtle GPCR adaptor affinities at the cellular base, while integrating fractal biomarkers for early detection across physiological hierarchies [165,166]. If indeed cells perceive and plot through GPCR adaptor-orchestrated networks, then so do organisms; ignoring these fractal threads courts therapeutic pipeline fragmentation but harnessing them could reforge biology’s boldest bias: evolution’s anticipatory artifice, now weaponized against its own unraveling. This provocative hypothesis of multi-scale biological influence, however, requires empirical testing through multiscale modeling and fractal biomarker analyses.

## 15. Conclusions

Cellular intelligence (CI)—encompassing sensory perception, reactive and adaptive responses, and memory-like processes—is a paradigm shift in understanding cellular resilience and disease. Stress-responsive GPCR networks, interconnected by signaling adaptors like β-arrestins and GRKs, serve as the molecular scaffold for this intelligence, orchestrating the detection, processing, and retention of environmental stressors. Dysregulation of these networks drives chronic diseases, from cancer to metabolic disorders, highlighting their therapeutic potential. Advances in omics, systems biology, and pharmacology will deepen our understanding of GPCR-mediated cellular intelligence, paving the way for precision interventions that restore adaptive responses and mitigate pathological outcomes. This perspective underscores the cell as an intelligent, GPCR-driven entity, redefining the boundaries of cellular biology and medicine.

## Data Availability

No new data were created or analyzed in this study. Data sharing is not applicable to this article.
